# Handling Missing Race and Ethnicity in an EHR-Based Study Through Integration of Individual Measures and Neighborhood Sociodemographic and Socioeconomic Measures

**DOI:** 10.3390/microorganisms14030662

**Published:** 2026-03-14

**Authors:** Chaohua Li, Abdolreza Mosaddegh, Lilly Immergluck, Samuel Owusu, Sadia Firoza Chowdhury, Peter Baltrus

**Affiliations:** 1 National Center for Primary Care, Morehouse School of Medicine, Atlanta, GA 30310, USA; cli@msm.edu (C.L.); sowusu@msm.edu (S.O.); 2 Department of Multidisciplinary Engineering, Northeastern University, Oakland Campus, Oakland, CA 94613, USA; a.mosaddegh@northeastern.edu; 3 Department of Pediatrics, University of Chicago, Chicago, IL 60637, USA; limmergluck1@uchicago.edu (L.I.); sadiafiroza.chowdhury@bsd.uchicago.edu (S.F.C.); 4 Department of Community Health and Preventive Medicine, Morehouse School of Medicine, Atlanta, GA 30310, USA

**Keywords:** imputation, electronic health records, pediatric health, race and ethnicity, missing data, machine learning, random forest, k-nearest neighbors, random forest

## Abstract

Race and ethnicity are frequently missing in electronic health records (EHRs), where excluding these records can bias pediatric research and disparity estimates. Imputing missing values may reduce this bias but can perform unevenly across methods and subgroups, especially for smaller or heterogeneous categories. We compared four approaches—logistic regression, random forest, k-nearest neighbors (KNN), and multiple imputation by chained equations (MICE)—to impute missing race (Black/White/Other) and ethnicity (Hispanic/Non-Hispanic) using individual- and census-tract-level sociodemographic measures. We analyzed 5309 children (<19 years) treated for Staphylococcus aureus infections at two pediatric hospitals in metropolitan Atlanta (2002–2015). The performance was evaluated on a held-out test set (n = 554) using accuracy and weighted F1. For race, logistic regression and KNN performed best (accuracy/weighted F1: 0.838/0.822 and 0.839/0.823), followed by random forest (0.798/0.787), with MICE being the lowest (0.736/0.743). For ethnicity, KNN achieved the highest accuracy (0.912) and random forest the highest weighted F1 (0.895) (logistic regression 0.901/0.876; random forest 0.904/0.895; KNN 0.912/0.887; MICE 0.866/0.864). Performance was the lowest for Hispanic ethnicity and the “Other” race category, consistent with the class imbalance. Imputation performance depends on the demographic attribute and modeling approach; subgroup-specific evaluation is essential when imputing race and ethnicity in pediatric EHR research.

## 1. Introduction

Many biomedical datasets, such as EHRs, have missing or low-quality demographic fields [1]. Demographic attributes, such as age, sex, race, ethnicity, and socioeconomic status, are critical variables in healthcare analytics that influence the treatment response [2]. However, these attributes are frequently missing, incomplete, or inaccurately recorded in EHRs and other medical data sources due to patient non-disclosure and inconsistent data collection [3]. Missing demographic data can bias statistical analyses and reduce the model performance in clinical systems [4]. As a result, imputing demographic attributes has become an important research area at the intersection of healthcare and machine learning.

Most studies focus on imputing sex, age, race and ethnicity, as these variables are commonly missing and strongly associated with clinical outcomes. Sex and age can often be inferred with relatively high accuracy using physiological or diagnostic characteristics [5]. In contrast, race, ethnicity and socioeconomic variables (e.g., insurance status) are more challenging due to their weaker associations with medical features [6]. Importantly, the accurate imputation of demographic attributes, particularly race and ethnicity, carries greater ethical and methodological significance than many other variables, as misclassification may reinforce structural biases or stereotypes embedded in healthcare data [7].

In recent years, traditional statistical imputation techniques have been increasingly replaced by machine learning approaches that employ high-dimensional clinical and contextual data more effectively [8]. Early work on demographic imputation mostly employed rule-based systems and statistical methods, including mean or mode imputation [9]. For example, age may be inferred from birthdate variables, while sex has typically been inferred using laboratory tests or diagnostic features.

Most traditional statistical methods assume linear relationships or specific data distributions and struggle to model complex nonlinear features in high-dimensional EHR data [10]. As EHR datasets grow in scale and complexity, these approaches have difficulty scaling. This motivates the adoption of machine learning techniques for demographic imputation.

Many studies on demographic imputation employ supervised classification or regression methods in which known demographic attributes are used as labels during model training, and predictions are generated for records with missing values [11]. Regression-based models remain popular due to their interpretability and computational efficiency, especially in clinical contexts [12]. However, their performance may degrade when relationships between predictors and demographic variables are highly nonlinear [13].

Some work uses multiple imputation (e.g., MICE) by modeling missing race and ethnicity from available individual-level variables [11]. However, multiple imputation depend heavily on the missingness mechanism (MAR vs. MNAR) and the availability of correlations between variables [14].

Tree-based ensemble methods such as random forests and boosting models have been widely used because they can model nonlinear relationships without strong distributional assumptions. Several studies suggest that random forests outperform linear models for imputing race and ethnicity from clinical and geographic features, especially in large datasets [6]. Moreover, deep learning approaches have also been used for demographic imputation, especially on large-scale or multimodal datasets [15]. However, due to the black-box nature of these models, employing them for imputing demographic attributes reduces the interpretability and transparency, which may lead to bias and lack of clinical trustworthiness [16].

Some studies combine machine learning models with proxy variables (e.g., surnames and geographic information) to improve race and ethnicity imputations [17]. This family of methods, including Bayesian Improved Surname Geocoding (BISG), exploits correlations between demographic attributes and geographic neighborhoods or name distributions. Extensions include adding first name information and other predictors (e.g., insurance coverage type) to improve the accuracy [18]. Recent studies on healthcare datasets suggest that these methods remain common because they require limited features (often just name and ZIP code), but their validity depends on the context [6]. A key limitation of BISG-based approaches is their dependence on geographically aggregated data, which may be sparse or unstable for minority populations in small geographic areas. In addition, applying these methods often requires the use of identifiable individual-level data, raising considerations related to data availability, governance, and compliance with privacy standards.

Because race and ethnicity are social constructs shaped by structural and historical forces, imputing these fields from EHRs and neighborhood context requires particular care. Imputation can reduce the bias from complete-case analysis, but it can also introduce misclassification that disproportionately affects minoritized groups, potentially reinforcing structural inequities if used uncritically. Due to these characteristics, one important aspect of imputation in clinical settings is the interpretability and transparency of the imputation method. While black-box and deep learning models can model complex relationships, the traceability and transparency provided by simpler white-box methods are also important for demographic imputation in clinical practice. However, it remains unclear whether employing more complex models can meaningfully improve the performance enough to justify the reduced transparency.

In this study, we aimed to compare four commonly used imputation approaches, including white-box methods like logistic regression and k-nearest neighbors, with more complex methods like random forest and multiple imputation by chained equations, for imputing missing race (Black/White/Other) and ethnicity (Hispanic/Non-Hispanic) in a pediatric electronic health record cohort. We treat imputed race and ethnicity as analytical proxies, not replacements for self-identified information, and emphasize transparency, subgroup-specific evaluation, and governance safeguards when such methods are used. Using individual- and census-tract–level sociodemographic information, we evaluated the performance using the overall accuracy and the weighted F1 score.

## 2. Materials and Methods

This study was based on data from a project that enrolled children with *Staphylococcus aureus* infections treated at two free standing pediatric hospitals, which are a part of Children’s Healthcare of Atlanta system (Egleston Children’s Hospital (ECH) and Scottish Rite Children’s Hospital (SRCH)) between 1 January 2002 and 31 December 2015. Demographic data and clinical data were obtained from the electronic health record (EHR). Antimicrobial susceptibility testing (AST) results were collected from routine hospital clinical microbiology laboratory reports. Eligible cases included unique patient-years with community-onset skin and soft tissue infection (CO-SSTI), age younger than 19 years, a valid georeferenced address within the 20-county Atlanta metropolitan statistical area (MSA) and showing resistance to at least one of the eight antibiotics based on AST results. The study was approved by the Institutional Review Boards of participating hospitals and affiliated academic institutions. Individual-level data used in this study included gender, age, and insurance type.

Census-tract level data was obtained from the 2006–2010 American Community Survey 5-Year Data [19], including the percentages of White/Black/Hispanic populations, percentage population less than 19 years old, percentage of population ≥3 years enrolled in K–12 education, percentage of population ≥3 years enrolled in nursery or preschool education, percentage with a high school diploma, percentage with a bachelor’s degree, percentage with a graduate or professional degree, percentage of the population in the labor force, median annual household income, median house value, percentage of crowded households (defined as housing unit with more than one person per room), percentage of population below federal poverty level, and Gini Index (a measure of income inequality from perfect equality 0, where everyone receives the same income, to perfect inequality 1, where a single person receives the total income of the community). Census tract level data was merged to individual-level data using Federal Information Processing Standard codes (FIPS codes).

To examine the association between the observed measures with the missingness in the race and ethnicity, we compared individual-level and census-tract-level measures between subjects with and without missing race or ethnicity, respectively. Chi-square tests were used for categorical measures and *t*-tests were used for continuous variables.

We tested four statistical/machine learning methods on the performance of imputing race and ethnicity: (1) multi-variable logistic regression, (2) random forest model, (3) K-nearest neighbors (KNN) model, and (4) multiple imputation by chained equations (MICE) model. For each method, all individual-level and census tract-level measures were included. To accommodate cases where race and ethnicity were missing at the same time, race was not used as a predictor to impute ethnicity and vice versa.

For the purpose of evaluating imputation performance, we restricted the analytic dataset used to train and test the models to patients with complete race and ethnicity and all other individual-level and census-tract-level measures. For this subset of data, we reserved 15% of the data as the test set (for evaluating the performance of imputation) and used the rest of the 85% to train the three imputation models (logistic regression, random forest, and KNN). To evaluate MICE on a held-out test set without label leakage, we combined training and test predictors, set the test-set race and ethnicity to missing, and then applied MICE. This mirrors the typical use of MICE to create a completed dataset while ensuring that true test labels were not used in model fitting.

Specifications for the models were as follows: (1) We fit a binary logistic regression model to impute ethnicity and a multinomial logistic regression model to impute race, using the ‘stats’ package (version 4.3.2) and ‘nnet’ package (version 7.3-19) in R (version 4.5.2), respectively. (2) When random forest was used for imputation, the model was fit using 500 trees, with five variables randomly sampled at each split and a minimum terminal node size of one. These values are commonly used in random forest implementations and were further confirmed through five-fold cross-validation to optimize the imputation performance; analyses were conducted using the ‘randomForest’ package (version 4.7-1.1) in R. (3) We implemented the KNN classifier in which predictions were based on the 10 nearest training observations (selected based on five-fold cross-validation). Euclidean distance was used to define similarity, and classification was performed by majority vote among neighbors. All numeric predictors were standardized, and dummy variables were created for categorical predictors prior to model fitting. Analyses were conducted using the ‘knn’ function from the ‘class’ package (version 7.3-22) in R. (4) Missing race and ethnicity values were imputed using MICE with variable-specific models. Race was imputed using multinomial logistic regression and ethnicity using binary logistic regression. A single imputed dataset was generated with 10 iterations of the imputation algorithm. Because the imputed outcomes were used for comparison with other models rather than for parameter inference, only a single imputed dataset was used. MICE was conducted within a fully conditional specification framework and under the assumption that the outcomes were missing at random. MICE was conducted using the ‘mice’ package (version 3.16.0) in R.

We used two metrics to evaluate the performance of the four imputation models on the test set: accuracy and weighted F1 score. Accuracy equals the proportion of observations that are correctly classified by a prediction model and can be calculated using the following formula:

$$Accuracy=\frac{Number\;of\;correct\;predictions}{Total\;number\;of\;predictions}$$

The weighted F1 score was defined as the class-frequency-weighted average of class-specific F1 scores, where each F1 score is the harmonic mean of precision and recall. This metric was chosen to account for class imbalance and to balance false positive and false negative errors, which are not adequately captured by the overall accuracy [20]. The detailed process used to calculate the weighted F1 score can be found in another study [21].

In addition to the accuracy and weighted F1, we also evaluated the sensitivity, specificity, positive predictive value (PPV), and negative predictive value (NPV) as supplemental measures to reflect performance under class imbalance. For ethnicity, metrics were calculated using a binary “positive class” (Hispanic). For race (multi-class), we calculated class-specific metrics using a one-vs.-rest approach for each category (Black, White, Other).

## 3. Results

We identified 5309 children who were younger than 19 years, treated for *S. aureus* infections at ECH and SRCH hospitals between 1 January 2002 and 31 December 2015, resided within the 20-county Atlanta metropolitan statistical area, and showed resistance to at least one of the eight antibiotics based on AST results. In this sample of children, 196 (3.7%) had a missing race. At the individual level, those with a missing race were featured by a higher percentage of using public insurance plan (71.4% vs. 58.6%) and a lower percentage of being older than 12 years (8.7% vs. 15.1%); at the census tract level, children with missing race resided in places with a lower percentage of Black population (27.5% vs. 42.4%), a higher percentage of Hispanic population (25.0% vs. 10.2%), a lower percentage with a high school diploma (23.8% vs. 27.2%), a higher percentage in the labor force (74.3% vs. 70.9%), a higher median house value ($213,374 vs. $195,274), a higher proportion of a crowded household (4.9% vs. 2.7%), a higher percentage living below the federal poverty level (22.0% vs. 17.3%), and a higher Gini Index (0.408 vs. 0.398) (Table 1).

There were 1608 out of 5309 (30.3%) children that had a missing ethnicity. At the individual level, children with a missing ethnicity were more likely to be female (54.7% vs. 49.7%) and use a public insurance plan (67.0% vs. 55.7%). Marked differences were also observed for most of the census-tract-level measures. Children with missing ethnicity resided in tracts with significantly lower proportions of White residents (31.5% vs. 53.9%) and higher proportions of Black residents (57.1% vs. 35.2%) compared with those without a missing ethnicity. No significant differences were observed in the proportion of residents younger than 19 years (*p* = 0.604) or labor force participation (*p* = 0.237). Socioeconomic measures at the census tract level consistently reflected a greater disadvantage among children with missing ethnicity. These children lived in tracts with lower educational attainment, as indicated by lower proportions of residents with bachelor’s degrees (18.4% vs. 21.2%) and graduate or professional degrees (9.6% vs. 11.0%). Additionally, census tracts where children with missing ethnicity resided had a significantly lower median annual household income ($50,204 vs. $61,326), lower median house values ($174,866 vs. $205,110), higher household crowding (3.15% vs. 2.61%), higher poverty rates (19.2% vs. 16.8%), and greater income inequality as measured by the Gini Index (0.404 vs. 0.395; all *p* < 0.001) (Table 2).

The race imputation performance was evaluated on the same test dataset using the accuracy and weighted F1 score (Table 3). Across the methods, the overall performance for race imputation was the highest for KNN and logistic regression (accuracy 0.839 and 0.838; weighted F1 0.823 and 0.822), followed by random forest (0.798; 0.787), with MICE performing the lowest (0.736; 0.743). However, the class-specific performance varied substantially. In the one-vs.-rest analyses (Table S1), sensitivity and PPV were generally strong for the Black and White categories, but the performance for the “Other” race category was consistently poor (sensitivity 0.000–0.095; PPV 0.000–0.125), indicating that the high overall accuracy partly reflected the correct classification of the majority classes rather than reliable identification of this rare and heterogeneous group.

The imputation performance for the ethnicity classification was evaluated on a held-out test dataset (n = 554) using the accuracy and weighted F1 score (Table 4). Among the four approaches, the KNN method achieved the highest overall accuracy (0.912), followed by random forest (0.904) and logistic regression (0.901). The MICE approach showed a lower overall accuracy (0.866) relative to the machine learning-based methods. The weighted F1 scores exhibited a similar pattern. Random forest yielded the highest weighted F1 score (0.895), indicating the best balance between precision and recall across both ethnic groups. KNN and logistic regression also performed well, with weighted F1 scores of 0.887 and 0.876, respectively. MICE had the lowest weighted F1 score (0.864). In the supplemental diagnostic metrics (Table S1), the specificity and NPV were high across the methods (specificity 0.930–0.992; NPV 0.914–0.929), whereas the sensitivity for the Hispanic ethnicity was comparatively low (0.164–0.327), consistent with the smaller size of the Hispanic class and the tendency for Hispanic cases to be misclassified as Non-Hispanic.

## 4. Discussion

In this study, we compared the performance of four commonly used statistical and machine learning approaches for imputing missing race and ethnicity in pediatric EHR data, integrating individual-level characteristics with neighborhood sociodemographic and socioeconomic measures. We observed considerable missingness in both race and ethnicity, which is consistent with prior studies revealing gaps in EHR demographic data quality [10]. When imputing ethnicity, KNN and random forest models achieved the highest accuracy and weighted F1 scores, with random forest providing the best balance between precision and recall for Hispanic classification. For race imputation, the simpler methods, i.e., logistic regression and KNN, performed comparably or better than random forest, suggesting that added model complexity does not uniformly translate into improved performance. In general, our findings demonstrate that the ideal accuracy of race and ethnicity imputation in EHR data can be achieved by integrating individual-level and neighborhood-level measures and highlight the importance of method selection based on specific demographic attributes, population characteristics, and evaluation metric of interest, rather than relying on a single default approach.

In our cohort of children with *S. aureus* infections, significant differences were observed between those with and without a missing race or ethnicity, indicating the missingness was not random. Missing race and ethnicity were both more common among children with public insurance plans and those residing in census tracts with higher poverty rates, greater household crowding, and higher income inequality. The strong associations between missingness and these characteristics suggest that race and ethnicity in pediatric EHR data are potentially missing at random conditional on available information, rather than missing completely at random. These findings imply that complete-case analyses in this setting may exacerbate bias, and model-based imputation methods that incorporate both individual-level and neighborhood-level covariates can mitigate information loss and reduce bias when missing-at-random assumptions are reasonable [4,14]. Despite ideal overall imputation performance for race and ethnicity, the imputation accuracy for certain subgroups, “Hispanic” in ethnicity and “Other” in race, was consistently low across the four methods. This is largely due to the limitation of using overall accuracy as the performance metric in the presence of considerable class imbalance and highlights the importance of complementary metrics, such as the weighted F1 score, that account for both precision and recall across imbalanced classes. Similar challenges have been documented in prior EHR-based imputation studies, where minority groups are more susceptible to misclassification, even when the aggregate performance appears acceptable [11].

Our findings generally align with and extend prior studies evaluating approaches for imputing race and ethnicity in healthcare data. Recent EHR-based studies have demonstrated that machine learning methods outperform traditional parametric or fully conditional imputation approaches when rich individual-level and contextual data are available [6,15]. Our results are consistent with this literature in showing a better performance of KNN and random forest relative to MICE for ethnicity imputation, while also suggesting that simpler models, such as logistic regression, can perform competitively for race imputation when the class structure is less complex. Imputation of race and ethnicity can affect minoritized groups, potentially reinforcing structural inequities if used uncritically. This highlights the importance of transparency and traceability in the imputation method. Our results show that while black-box methods reduce the traceability and transparency by adding complexity, they do not improve the accuracy in conventional datasets compared with simpler white-box models. However, black-box and deep learning models may be better suited to high-dimensional, multimodal datasets. This aspect can be further investigated in future imputation studies using more complex datasets.

Moreover, in contrast to approaches that rely primarily on proxy variables, such as surnames or limited geographic indicators, recent healthcare applications have emphasized the potential of combining neighborhood sociodemographic context with individual-level characteristics to improve imputation accuracy [18]. By applying and evaluating multiple methods within a unified pediatric EHR framework using the same predictors and evaluation metrics, this study contributes comparative evidence that method performance is context- and outcome-dependent, echoing recommendations that no single imputation strategy should be considered universally optimal across populations or demographic features.

Our study has several strengths, including the use of a pediatric EHR cohort spanning more than a decade, the integration of individual-level clinical information with census-tract-level sociodemographic and socioeconomic measures, and a head-to-head comparison of multiple imputation approaches using consistent predictors and evaluation metrics. Several limitations should also be acknowledged. First, this study included children with *S. aureus* infections from two pediatric hospitals within a single metropolitan region, which may limit the generalizability of the findings to the broader pediatric population, other healthcare systems, or different geographic settings. The documentation practices, missingness mechanisms, and the relationship between individual-level characteristics, neighborhood context, and race and ethnicity may differ in other regions, health systems, time periods, and clinical. Therefore, model performance and subgroup error profiles observed here may not directly generalize, highlighting the need for external validation in broader pediatric cohorts and in geographically diverse EHR settings. Second, imputation was based on the assumption that race and ethnicity were missing at random, though the possibility of missing not at random (MNAR) cannot be ruled out. Because missingness in race and ethnicity was associated with observed individual- and neighborhood-level characteristics, we treated the missingness mechanism as plausibly MAR conditional on these covariates. However, if missingness demonstrated MNAR (dependent on the true, unobserved race and ethnicity after conditioning on observed covariates), imputed values could be systematically biased, particularly for under-represented groups, potentially biasing downstream disparity analyses. We therefore interpret imputations as probabilistic proxies rather than replacements for self-reported race and ethnicity. Finally, limited sample sizes for “Hispanic” in ethnicity and “Other” in the race category likely constrained classification performance. Despite these limitations, the consistency of findings across imputation methods supports the robustness of the overall conclusions.

While imputing race and ethnicity can reduce bias from complete-case analysis when missingness is related to observed covariates, it can also introduce new biases through differential misclassification, particularly for smaller or heterogeneous categories (e.g., Hispanic ethnicity and “Other” race). Because race and ethnicity are social variables rather than biological traits, imputing them from proxies, such as neighborhood composition or socioeconomic context, risks reifying structural inequities embedded in historical and geographic patterns. We therefore recommend that imputed race and ethnicity be used primarily for research sensitivity analyses and bias assessment, not for individual-level clinical decision-making. When used, investigators should report subgroup-specific performance, quantify uncertainty, and evaluate whether conclusions about disparities are robust across complete-case and imputed analyses.

In conclusion, our study demonstrates that the performance of race and ethnicity imputation methods in pediatric EHR data depends on both the demographic attribute being imputed and the choice of modeling and evaluation metrics. Machine learning approaches integrating individual and neighborhood-level information can achieve strong performance. No single method consistently outperformed others across outcomes or subgroups. These findings underscore the importance of selecting imputation methods, reporting subgroup-specific performance, and using metrics that account for class imbalance. Improving the handling of missing race and ethnicity data is essential for producing valid and equitable pediatric health research, and future work should involve data from more diverse settings or broader geographic areas; external validation across health systems; and other emerging imputation methods, like deep learning and ensemble machine learning.

## Figures and Tables

**Table 1:** Distribution of individual level and census tract level sociodemographic measures by missingness of race.

	Missing Race		
	Missing	Not Missing	
Individual Level	%	%	*p*-Value
Total	196	5113	
Gender			0.809
Female	52.0	51.2	
Male	48.0	48.8	
Age			0.018
0–3 years	57.7	57.6	
4–12 years	33.7	27.3	
>12 years	8.7	15.1	
Insurance			0.002
Public	71.4	58.6	
Private	25.0	35.0	
Other	3.6	6.4	
Census Tract Level	Mean	Mean	*p*-Value
White, %	49.3	47	0.318
Black, %	27.5	42.4	<0.001
Hispanic, %	25	10.2	<0.001
<19 years, %	28.5	29.7	0.003
K-12th grades enrollment, %	68.2	69.8	0.053
Nursery to preschool enrollment, %	7.7	7.6	0.792
High school, %	23.8	27.2	<0.001
Bachelor, %	22.1	20.2	0.021
Graduate or professional, %	11.4	10.5	0.141
Labor force, %	74.3	70.9	<0.001
Median annual household income, $	54,820	58,077	0.08
Median house value, $	213,374	195,274	0.011
Household crowding, %	4.9	2.7	<0.001
Poverty%	22	17.3	<0.001
Gini Index	0.408	0.398	0.012

*p*-values were calculated using Chi-square tests for categorical measures and *t*-tests for continuous measures.

**Table 2:** Distribution of individual-level and census-tract-level sociodemographic measures by missingness of ethnicity.

	Missing Ethnicity		
	Missing	Not Missing	
Individual Level	%	%	*p*-Value
Total	1608	3701	
Gender			<0.001
Female	54.7	49.7	
Male	45.3	50.3	
Age			0.172
0–3 years	59.4	56.8	
4–12 years	26.8	27.9	
>12 years	13.8	15.3	
Insurance			<0.001
Public	67.0	55.7	
Private	25.1	38.8	
Other	7.9	5.5	
Census Tract Level	Mean	Mean	*p *-Value
White, %	31.49	53.9	<0.001
Black, %	57.14	35.17	<0.001
Hispanic, %	10.85	10.73	<0.001
<19 years, %	29.74	29.65	0.604
K-12th Grades enrollment, %	68.62	70.21	<0.001
Nursery to preschool enrollment, %	7.29	7.73	<0.001
High school, %	28.13	26.63	<0.001
Bachelor, %	18.37	21.16	<0.001
Graduate or professional, %	9.56	11	<0.001
Labor force, %	71.21	70.94	0.237
Median annual household income, $	50,204	61,326	<0.001
Median house value, $	174,866	205,110	<0.001
Household crowding, %	3.15	2.61	<0.001
Poverty%	19.18	16.79	<0.001
Gini Index	0.404	0.395	<0.001

*p*-values were calculated using Chi-square tests for categorical measures and *t*-tests for continuous measures.

**Table 3:** Prediction results of race (Black/White/Other) by four different methods.

Method		Predicted Race				Accuracy	Weighted F1 Score
	True Race	Black	White	Other	Total		
Logistic regression	Black	126	38	1	165	0.838	0.822
	White	29	338	1	368		
	Other	6	15	0	21		
	Total	161	391	2	554		
Random forest	Black	112	49	4	165	0.798	0.787
	White	36	329	3	368		
	Other	8	12	1	21		
	Total	156	390	8	554		
KNN	Black	125	39	1	165	0.839	0.823
	White	28	340	0	368		
	Other	6	15	0	21		
	Total	159	394	1	554		
MICE	Black	111	44	10	165	0.736	0.743
	White	55	295	18	368		
	Other	6	13	2	21		
	Total	172	352	30	554		

KNN, k-nearest neighbors; MICE, multiple imputation by chained equations.

**Table 4:** Prediction results of ethnicity (Hispanic/Non-Hispanic) by four different methods.

Method	True Ethnicity	Predicted Ethnicity			Accuracy	Weighted F1 Score
		Hispanic	Non-Hispanic	Total		
Logistic regression	Hispanic	9	46	55	0.901	0.876
	Non-Hispanic	9	490	499		
	Total	18	536	554		
Random forest	Hispanic	18	37	55	0.904	0.895
	Non-Hispanic	16	483	499		
	Total	34	520	554		
KNN	Hispanic	10	45	55	0.912	0.887
	Non-Hispanic	4	495	499		
	Total	14	540	554		
MICE	Hispanic	16	39	55	0.866	0.864
	Non-Hispanic	35	464	499		
	Total	51	503	554		

KNN, k-nearest neighbors; MICE, multiple imputation by chained equations.

## Data Availability

The datasets presented in this article are not readily available due to confidentiality issues. Requests to access the datasets should be directed to Peter Baltrus.

## Supplementary Materials

The following supporting information can be downloaded from https://www.mdpi.com/article/10.3390/microorganisms14030662/s1. Table S1: Class-specific diagnostic performance for ethnicity imputation (Hispanic vs. Non-Hispanic) and race imputation (Black/White/Other) in the held-out test set (n = 554).
