# Supplementary material for: Handling Missing Race and Ethnicity in an EHR-Based Study Through Integration of Individual Measures and Neighborhood Sociodemographic and Socioeconomic Measures
Source: Microorganisms. 2026 Mar 14;14(3):662. doi: 10.3390/microorganisms14030662 (PMC13028836; doi:10.3390/microorganisms14030662)
Supplement: Supplementary file 1 [file microorganisms-14-00662-s001.zip › microorganisms-4157545-supplementary.pdf]

Table S1. Class-specific diagnostic performance for ethnicity imputation (Hispanic vs Non-Hispanic) and race imputation (Black/White/Other) in the held-out test set (n = 554)

Method	Race	Sensitivity	Specificity	PPV	NPV
Logistic regression	Black	0.764	0.91	0.783	0.901
	White	0.918	0.715	0.864	0.816
	Other	0	0.996	0	0.962
Random forest	Black	0.679	0.887	0.718	0.867
	White	0.894	0.672	0.844	0.762
	Other	0.048	0.987	0.125	0.963
KNN	Black	0.758	0.913	0.786	0.899
	White	0.924	0.71	0.863	0.825
	Other	0	0.998	0	0.962
MICE	Black	0.673	0.843	0.645	0.859
	White	0.802	0.694	0.838	0.639
	Other	0.095	0.947	0.067	0.964
**Method**	**Ethnicity**	**Sensitivity**	**Specificity**	**PPV**	**NPV**
Logistic regression	Hispanic	0.164	0.982	0.5	0.914
Random forest	Hispanic	0.327	0.968	0.529	0.929
KNN	Hispanic	0.182	0.992	0.714	0.917
MICE	Hispanic	0.291	0.93	0.314	0.922

Sensitivity= TP/(TP+FN); specificity = TN/(TN+FP); positive predictive value (PPV) = TP/(TP+FP); negative predictive value (NPV) = TN/(TN+FN).

KNN, k-nearest neighbors; MICE, multiple imputation by chained equations.

Metrics were calculated using a one-vs-rest approach for each race category when imputing race; Hispanic was treated as the positive class when imputing ethnicity.
